# Langmuir Films of Perfluorinated Fatty Alcohols: Evidence of Spontaneous Formation of Solid Aggregates at Zero Surface Pressure and Very Low Surface Density

**DOI:** 10.3390/nano10112257

**Published:** 2020-11-14

**Authors:** Pedro Silva, Duarte Nova, Miguel Teixeira, Vitória Cardoso, Pedro Morgado, Bruno Nunes, Rogério Colaço, Marie-Claude Fauré, Philippe Fontaine, Michel Goldmann, Eduardo J. M. Filipe

**Affiliations:** 1 Centro de Química Estrutural and Departamento de Engenharia Química, Instituto Superior Técnico, Universidade de Lisboa, 1049-001 Lisboa, Portugal; pedro.ribeiro.silva@tecnico.ulisboa.pt (P.S.); duartenova1@gmail.com (D.N.); miguelcabritateixeira@gmail.com (M.T.); vitoria001@hotmail.com (V.C.); pm.elessar@gmail.com (P.M.); bruno.nunes@ist.utl.pt (B.N.); rogerio.colaco@tecnico.ulisboa.pt (R.C.); 2 Institut des NanoSciences de Paris, UMR 7588 CNRS Sorbonne Université, 4 Place Jussieu, 75252 Paris CEDEX 05, France; marie-claude.faure@insp.jussieu.fr (M.-C.F.); michel.goldmann@insp.upmc.fr (M.G.); 3 Synchrotron SOLEIL, L’Orme des Merisiers, Saint Aubin, BP48 91192 Gif sur Yvette CEDEX, France; philippe.fontaine@synchrotron-soleil.fr; 4 Faculté des Sciences Fondamentales et Biomédicales, Université de Paris, 45 rue des Saints-Pères, 75006 Paris, France

**Keywords:** perfluorinated alcohols, Langmuir films, GIXD

## Abstract

In this work, Langmuir films of two highly fluorinated fatty alcohols, CF_3_(CF_2_)_12_CH_2_OH (F14OH) and CF_3_(CF_2_)_16_CH_2_OH (F18OH), were studied. Atomic Force Microscopy (AFM) images of the films transferred at zero surface pressure and low surface density onto the surface of silicon wafers by the Langmuir-Blodgett technique revealed, for the first time, the existence of solid-like domains with well-defined mostly hexagonal (starry) shapes in the case of F18OH, and with an entangled structure of threads in the case of F14OH. A (20:80) molar mixture of the two alcohols displayed a surprising combination of the two patterns: hexagonal domains surrounded by zigzagging threads, clearly demonstrating that the two alcohols segregate during the 2D crystallization process. Grazing Incidence X-ray Diffraction (GIXD) measurements confirmed that the molecules of both alcohols organize in 2D hexagonal lattices. Atomistic Molecular Dynamics (MD) simulations provide a visualization of the structure of the domains and allow a molecular-level interpretation of the experimental observations. The simulation results clearly showed that perfluorinated alcohols have an intrinsic tendency to aggregate, even at very low surface density. The formed domains are highly organized compared to those of hydrogenated alcohols with similar chain length. Very probably, this tendency is a consequence of the characteristic stiffness of the perfluorinated chains. The diffraction spectrum calculated from the simulation trajectories compares favorably with the experimental spectra, fully validating the simulations and the proposed interpretation. The present results highlight for the first time an inherent tendency of perfluorinated chains to aggregate, even at very low surface density, forming highly organized 2D structures. We believe these findings are important to fully understand related phenomena, such as the formation of hemi-micelles of semifluorinated alkanes at the surface of water and the 2D segregation in mixed Langmuir films of hydrogenated and fluorinated fatty acids.

## 1. Introduction

Highly fluorinated compounds display remarkable physicochemical properties that make them attractive for a myriad of applications, particularly in the biomedical field. Given their inertness and biocompatibility, they can be used as oxygen carriers, additives in blood-substituting formulations [1,2], drug carriers [3,4] and as ultrasound imaging contrast agents [5,6].

The interfacial properties of fluorinated amphiphiles are well known and noteworthy. Surfaces formed by perfluorinated chains, of which polytetrafluoroethylene (Teflon^®^) is the typical example, are simultaneously hydrophobic and lipophobic. The surface tension of perfluoroalkanes is on average half of that of alkanes with similar chain length. Consequently, perfluorinated surfactants in aqueous solution are able to reduce the surface tension of water down to much lower values than their hydrogenated analogues. Fluorinated surfactants are thus used in numerous formulations, as fire retardants, water and dirt repellents, among many others [7,8].

Langmuir and Langmuir-Blodgett films (LB) are useful and well-established model systems in the field of bi-dimensional fundamental physics. The organization and thermodynamic properties of LB films of hydrogenated fatty acids, alcohols, phospholipids, etc. are well established [9]. On the contrary, studies of Langmuir films of fluorinated amphiphiles have been less systematic and scarcer. Nonetheless, there have been important efforts to describe and interpret phase transitions and molecular organization in Langmuir films of several perfluorinated acids, alcohols and phospholipids [10,11,12,13,14], as well as amphiphiles with more complex molecular structure such as fluorinated bolaamphiphiles [15,16].

The main purpose of this work is to contribute to understanding the self-assembling of perfluorinated fatty alcohols, in particular in the very low surface density region. Following our recent paper on perfluorinated fatty acids [12], a combined experimental and computational methodology is used, bringing together experimental data (thermodynamic, atomic force microscopy (AFM), grazing incidence X-ray diffraction (GIXD)) and atomistic molecular dynamics simulations results.

In terms of molecular structure, the substitution of hydrogen atoms for fluorine atoms in a carbon chain has a number of important consequences. Given the larger radius and mass of fluorine compared to hydrogen, perfluorinated chains have a wider cross-section (0.283 nm^2^ compared to 0.185 nm^2^ [17]) and consequently higher molar volumes and densities than hydrogenated chains with the same number of carbon atoms. As for molecular conformations, perfluorinated chains are known to display a characteristic helical conformation, unlike hydrogenated chains that tend to be in their all-trans planar form [18]. Moreover, in contrast with the flexible character of hydrogenated chains, perfluorinated chains are known to be rigid, as a consequence of their much higher energy barrier to internal rotation [19]. All together, these features have a number of thermodynamic consequences. Perfluorinated alcohols for example, have higher melting temperatures [20,21], and are more volatile (lower boiling temperatures) than hydrogenated alcohols, thus displaying a smaller liquid range. In other words, solidification is easier for perfluorinated substances.

An additional important characteristic of perfluorinated chains is their unexpected and still unexplained weak interactions with hydrogenated chains. For example, mixtures of alkanes and perfluoroalkanes display regions of liquid–liquid immiscibility and large positive excess enthalpies and volumes. These observations are interpreted as sign of weak intermolecular dispersion forces between perfluorinated and hydrogenated chains [22,23,24,25,26,27,28]. Anomalies in the transport [29] and surface [30] properties of mixtures of alkanes and perfluoroalkanes have also been reported. At the molecular scale, nano-segregation has been demonstrated in (*n*-hexane + *n*-perfluorohexane) mixtures, above the upper critical solution temperature (UCST) [31,32,33]. In addition, it has been recently demonstrated that the presence of fluorinated groups induces conformational changes in hydrogenated chains, leading to more globular arrangements, increasing the proportion of gauche conformations [34]. This coiling effect was also detected in mixtures of hydrogenated and fluorinated alcohols [35].

This intrinsic “antipathy” between hydrogenated and perfluorinated substances has been the basis for a number of studies on the segregation and 2D phase-separation in Langmuir films of mixtures of hydrogenated and perfluorinated carboxylic acids [36,37,38,39,40,41,42] and alcohols [43,44].

Finally, molecules formed by the antagonistic hydrogenated and perfluorinated moieties covalently bonded together form a special type of amphiphiles, perfluoroalkylalkanes (PFAA), often called *primitive surfactants*, due to the lack of hydrophilic head group. PFAAs are known to form highly ordered arrays of hemi-micelles at the surface of water [45,46,47], that can be transferred to the surface of solid substrates and used as templates for surface nano-patterning [48]. The principles governing the formation of such hemi-micelles have only recently been explained [49]. Langmuir films of partially fluorinated carboxylic acids also display nano domains of remarkable morphology [50,51,52].

In this work, we studied the Langmuir films of two highly fluorinated fatty alcohols, CF_3_(CF_2_)_12_CH_2_OH (F14OH) and CF_3_(CF_2_)_16_CH_2_OH (F18OH) and a (20:80) molar mixture of the two alcohols.

The films were transferred onto the surface of silicon wafers by the Langmuir-Blodgett technique and analyzed by atomic force microscopy (AFM). The AFM images of the films transferred at very low surface pressure, revealed the existence of solid-like domains with well-defined mostly hexagonal (starry) shapes in the case of F18OH, and with a complex entangled structure in the case of F14OH. The mixture of the two alcohols displayed a surprising combination of the two patterns, namely hexagonal domains surrounded by zigzagging threads, demonstrating the existence of 2D segregation of the two molecules.

The crystalline nature of the domains was confirmed by Grazing Incidence X-ray Diffraction (GIXD) performed on the SIRIUS beamline at the SOLEIL synchrotron in Saint-Aubin, France. Both alcohols display 2D hexagonal lattices.

Finally, atomistic Molecular Dynamics (MD) simulations were performed to provide a molecular-level interpretation of the experimental results and a visualization of the structure of the monolayers. The diffraction pattern calculated from the simulation results reproduces the experimental X-ray spectrum, fully validating the simulation’s methodology and force field. The simulation results clearly show that the fluorinated alcohols have an intrinsic tendency to aggregate, even at very low surface density, forming clusters, which are highly organized compared to those of hydrogenated alcohols with similar chain length. Very probably, this tendency is a consequence of the characteristic stiffness of the perfluorinated chains.

The present results highlight for the first time an inherent tendency of perfluorinated chains to aggregate, even at very low surface density, forming highly organized 2D structures. We believe these findings will be important to fully understand related phenomena, such as the 2D segregation in mixed Langmuir films of hydrogenated and fluorinated fatty acids and the formation of hemi-micelles of perfluoroalkylalkanes at the surface of water.

## 2. Materials and Methods

### 2.1. Molecules and Sample Preparation

The fluorinated alcohols 1*H*,1*H*-perfluorotetradecan-1-ol (F14OH) and 1*H*,1*H*-perfluorooctadecan-1-ol (F18OH) were acquired from Fluorochem, Hadfield, UK, both with a stated purity of 95%, and used as received. Stock solutions with concentrations ranging from 0.2 mg/mL to 1 mg/mL of these alcohols were prepared by dissolving appropriate amounts of the alcohols in a spreading solvent made of 1,1,2-trichlorotrifluoroethane (Aldrich, purity >99.9%) and perfluorohexane (Aldrich, purity 99%) in a 9:1 (v:v) proportion. The solutions were immersed in an ultrasound bath for at least 10 min to help dissolve the alcohol. Perfluorohexane was stored with 3 Å molecular sieves, and 1,1,2-trichlorotrifluoroethane was purified as described by Armarego and Perrin [53] and kept under nitrogen atmosphere.

The Langmuir films were prepared using water from a Milli-Q Millipore system (Darmstadt, Germany; resistivity 18.2 MΩ cm) as the subphase in a NIMA Langmuir trough type 601 (Espoo, Finland) and in a homemade trough for GIXD measurements, with 600 cm^2^ and 700 cm^2^, respectively. The surface pressure was measured by the Wilhelmy plate method, using a filter paper plate. The temperature of the subphase was controlled by a water circulation bath and measured using a submerged platinum resistance thermometer. The isotherms were recorded in continuous compression mode at a constant rate of between 0.03 and 0.09 nm^2^/molecule/min, after a waiting period of at least 10 min to allow the spreading solvent to evaporate.

The Langmuir-Blodgett (LB) films were prepared by vertically immersing a silicon wafer (~8 cm^2^) in the water subphase before spreading, compressing the molecular film up to the desired surface pressure or molecular area, and then extracting the wafer at constant surface pressure at a speed of 3 mm/min. The prepared LB films were left to dry in the air and stored in a protective dust-proof case before being imaged by AFM. Prior to being used, the silicon wafers were first scrubbed with an aqueous solution of detergent, abundantly rinsed with Millipore water, submerged in chromic mixture for at least 30 min and then rinsed and stored in Millipore water afterwards.

### 2.2. Atomic Force Microscopy (AFM)

AFM topography images were collected using a Nanosurf Easyscan 2 Atomic Force Microscope system (Liestal, Switzerland) operating in tapping mode, using a NANOSENSOR PPP-NCLAuD probe (Neuchatel, Switzerland) with a resonance frequency in the range of 204–497 kHz. Square images with sides ranging from 3 µm to 100 µm were obtained in air with a resolution of 256 lines per image and 256 pixels per line. The images were treated (polynomial background and line removal) using the open-source software Gwyddion (version 2.56, 2020) [54].

### 2.3. Grazing Incidence X-ray Diffraction (GIXD)

GIXD experiments were performed on the SIRIUS beamline at the SOLEIL synchrotron source in Saint-Aubin, France. The details and the optics of the facility are described in reference [55]. The energy of the incident X-ray was 8 keV (*λ* = 0.155 nm) and the incident beamsize was 0.1 × 2 mm^2^ (*V* × *H*). The surface was illuminated at an incident angle of 2.00 mrad, below the critical angle of the air–water interface (2.8 mrad at 8 keV). Thus, the incident wave was almost totally reflected, while the refracted wave became evanescent, leading to a probed thickness of about 5 nm beneath the interface. The scattered intensity was collected with very low noise, using a 2D Pilatus detector fixed on the 2-axis detector arm of the beamline’s diffractometer. A Soller slit collimator is positioned in front of the detector, leading to an in-plane wave vector resolution of 0.02 nm^−1^ in the Q_xy_ range recorded.

### 2.4. Simulation Details

Molecular Dynamics simulations were conducted for a series of films of 1*H*,1*H*-perfluorinated alcohols, bearing linear chains of 12, 14, 16 and 18 carbon (C) atoms (F12OH, F14OH, F16OH and F18OH) at the vacuum-water interface, using a force field based on the Optimised Potentials for Liquid Simulations (OPLS) framework [56]. As in our previous study of fluorinated alcohols, the terminal –CF_2_CH_2_OH segment was modelled after the work of Duffy on trifluoroethanol [57,58], adjusting the partial charge of the fluorinated carbon to keep the electrical neutrality of the molecule. The model for the remainder of the perfluorinated tail is based on the parameters described by Watkins and Jorgensen [59], with adjustments to the atomic partial charges and to the dispersive energy parameter (*ε*) of fluorine atoms to account for the long CF chains, as described in previous work [12]. The rigid 3-site SPC/E model was used for the water molecules [60]. Following the OPLS framework, geometric combination rules were used to calculate the heteronuclear LJ interaction parameters (*ε_ij_* and *σ_ij_*).

The simulations were constructed based on a tetragonal 9.1 × 9.1 × 20 nm^3^ simulation box containing 10,000 water molecules in slab geometry, equilibrated for 3 ns in the NVT ensemble before any further use. The dimensions of the box were chosen to ensure a sufficiently thick (~3.5 nm) water slab with two explicit liquid-vapor interfaces perpendicular to the z axis. Periodic boundary conditions were applied in all directions, the height of the box being sufficient to ensure the interaction of the periodic images of the system along the z direction were negligible. To generate a larger surface area, this initial box was replicated in the x and y directions, and an additional 1 ns simulation run was performed for equilibration; all subsequent simulated systems were built from these two equilibrated water slabs.

The initial configurations of the simulated molecular films were generated using the Packmol open-source software package (version 15.178, 2015) [61], by inserting alcohol molecules in the free space upon the water slabs obtained. For each system, an appropriate number of alcohol molecules in their minimum energy configuration was inserted, aligned with the z axis and with the hydroxyl group facing the surface. Typically, the fluorinated molecules were placed in a close packed cylinder configuration, in close proximity to the water slab. Simulation runs of at least 10 ns were performed in the NVT ensemble, with at least 2 ns discarded from analysis as equilibration time; no evolution of the system properties was identified during the production phase of the simulations. The open-source GROMACS simulation engine (version 5.0.7, 2015) [62,63] was used to run the simulations, using the leap-frog algorithm to solve the equations of motion in 2 fs timesteps. All bonds involving hydrogen atoms were constrained to their equilibrium positions using the LINCS algorithm [64]. The van der Waals and electrostatic interactions were calculated explicitly up to a 1.4 nm cut-off, and the Particle Mesh Ewald (PME) method was used to account for the same interactions at longer distances. All the simulations were conducted at 293.15 K, using the V-Rescale thermostat with a coupling constant of 0.5 ps [65].

The positions of the atoms were saved every 1 ps for subsequent analysis of the trajectory. All the snapshots and visual representations of the simulations were obtained using the Visual Molecular Dynamics (VMD) open-source software package (version 1.9.3, 2016) [66].

## 3. Results

### 3.1. Thermodynamic Study

**Figure 1:**
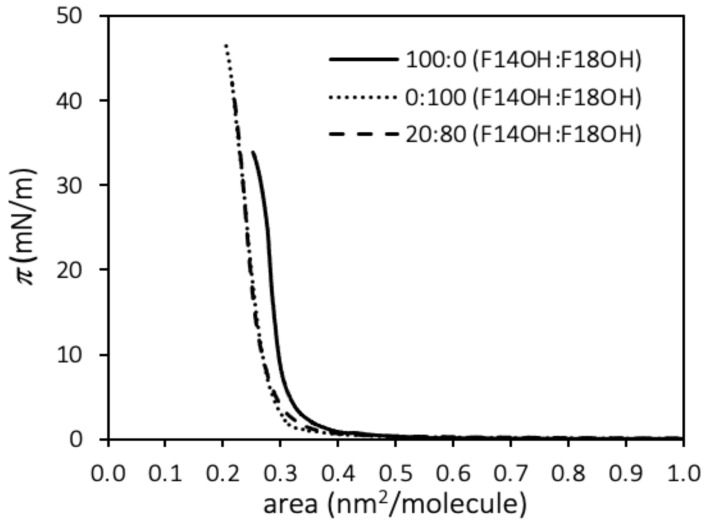
Surface pressure—molecular area (*π*–*A*) compression isotherms at 20 °C for monolayers of F14OH, F18OH and a 20:80 molar mixture of the two alcohols.

The surface pressure–Area per molecule (*π*–*A*) compression isotherms at 20 °C of monolayers of F18OH, F14OH and a 20:80 molar mixture of F14OH + F18OH are shown in Figure 1.

The isotherms obtained for the pure components compare well to other results reported in the literature for fluorinated alcohols [67,68]. The compression isotherms of both alcohols, starting at zero pressure and large areas per molecule (*A* > 0.9 nm^2^/molecule), display liftoff areas, *A*_0_, which are characteristic of perfluorinated chains (~0.3 nm^2^). The A_0_ values, obtained as usual from the extrapolation to zero pressure of the linear region of the isotherm before the film collapse, are reported in Table 1. The shorter alcohol, F14OH, displays a larger value than F18OH, which can be an indication of a lower cohesion of the condensed phase of F14OH, with a less efficient packing of its shorter fluorinated chains. In analogy with hydrogenated amphiphiles, the long horizontal plateau at zero surface pressure for *A* > *A*_0_, suggests coexistence (and a first order transition) between a gaseous phase and a condensed phase.

The isotherm obtained for the mixture of the two perfluorinated alcohols shows a similar behavior, following more closely the isotherm of F18OH. The limit area is similar, within the uncertainty, to that of F18OH; even though F18OH is the more abundant component of the mixture, the near coincidence of the isotherms and of the limit area may also be an indication that, when the film is very compressed, the longer F18OH molecules are able to increase the packing efficiency of the shorter F14OH particles.

### 3.2. Grazing Incidence X-ray Diffraction Results

**Figure 2:**
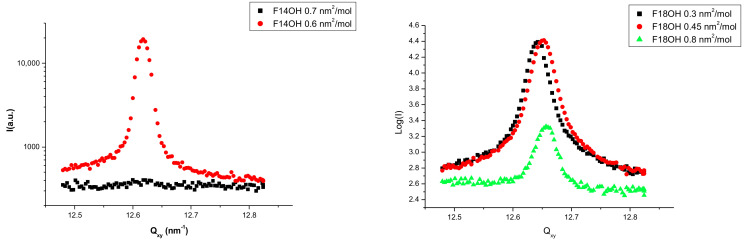
Diffraction spectra at 18 °C of the Langmuir films of F14OH (**left**) and F18OH (**right**) at different surface areas along the zero surface pressure plateau.

Langmuir films of the two pure alcohols (F14OH and F18OH) were studied by GIXD at different surface densities, along the zero pressure plateau. No diffraction signal could be detected at surface areas above 0.7 nm^2^/molecule for F14OH and above 0.8 nm^2^/molecule for F18OH. The obtained Q_z_-integrated spectra are presented in Figure 2. The positions and the full width at half maximum (FWHM) of the diffraction peaks, obtained from the fitting of Lorentzian peaks to the experimental data, are summarized in Table 2.

As can be seen, sharp diffraction peaks are observed for both compounds, confirming in both cases the existence of crystalline structures at the surface. These observations are interpreted as an indication of the tendency of the perfluorinated alcohols to spontaneously organize in 2D solid films even at these very low surface densities and zero surface pressure. As inferred from the *π*–*A* isotherms above, this solid phase coexists with a 2D gas phase. The crystalline organization can be quantified by the coherence length *l*_c_, calculated as 2π times the inverse of the FWHM of the peaks: *l*_c_(F14OH) > 320 nm and *l*_c_(F18OH) ~300 nm. From the position of the peaks and considering a hexagonal structure, areas of 0.286 nm^2^/molecule and 0.285 nm^2^/molecule are obtained for F14OH and F18OH, respectively, at zero surface pressure, close to the cross-sectional area of perfluorinated chains and consistent with a tight packing of the molecules in the Langmuir film.

**Figure 3:**
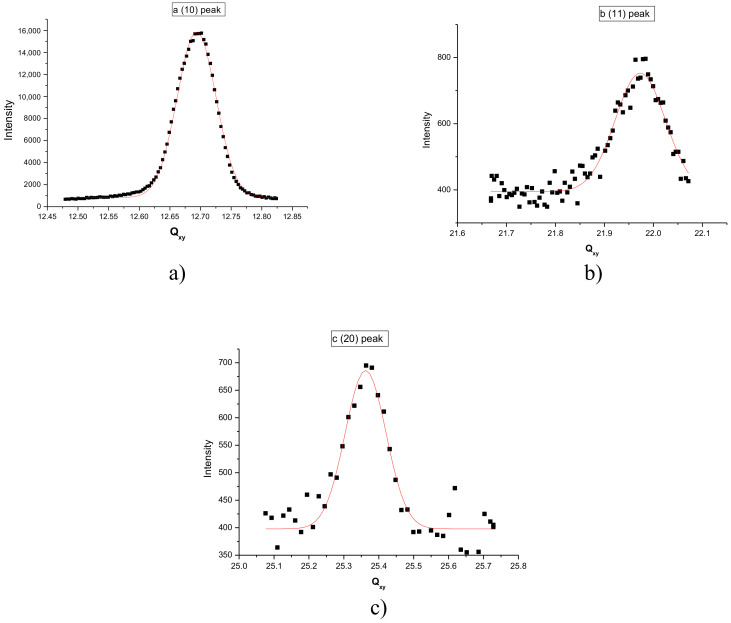
Q_z_ integrated diffraction spectrum at 18 °C and 15 mN/m of the Langmuir film of F18OH. (**a**) (10) peak; (**b**) (11) peak; (**c**) (20) peak.

The monolayer of F18OH was further studied by GIXD at 15 mN/m. The hexagonal structure of the LC phase was confirmed by measuring the (10), (11) and (20) peaks (Figure 3). The positions and the FWHM of the diffraction peaks, obtained from the fitting of Gaussian peaks to the experimental data, are summarized in Table 3 in perfect agreement with the hexagonal lattice. The maximum intensity of the rod localized in the diffraction plan ensures that the chains are vertical.

### 3.3. Atomic Force Microscopy Results

**Figure 4:**
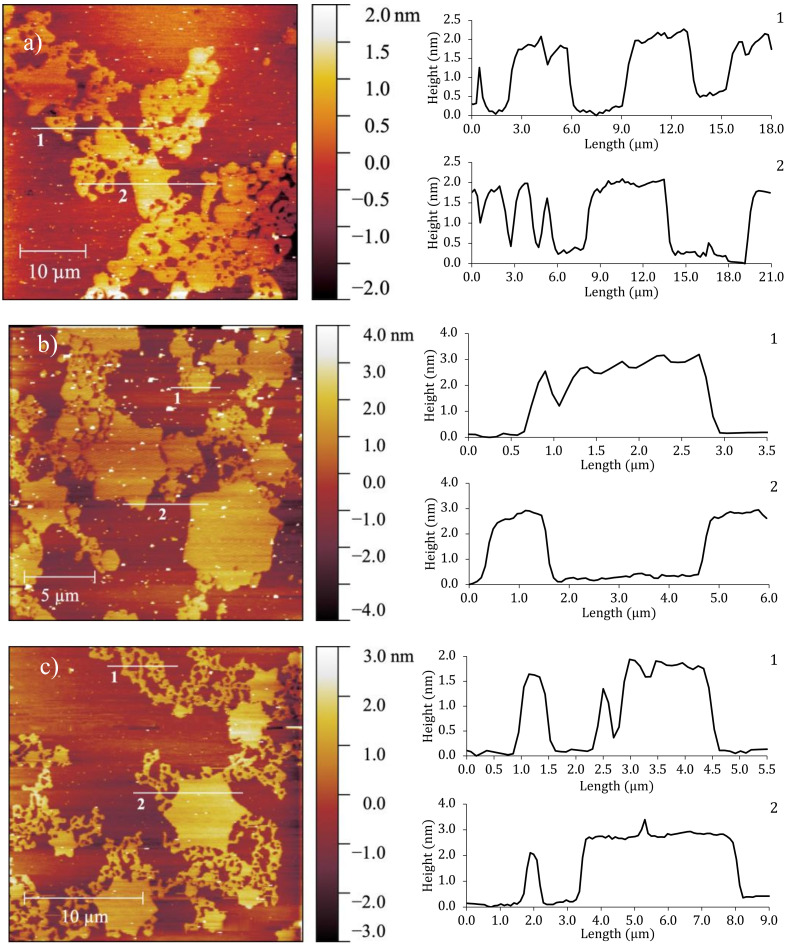
AFM height images of (**a**) F14OH at 0.7 nm^2^/molecule, (**b**) F18OH at 0.85 nm^2^/molecule and (**c**) (20:80) molar mixture of F14OH + F18OH at 0.8 nm^2^/molecule. The right panels show the height profiles along the numbered lines on the AFM images.

The monolayers were studied at low surface density where no GIXD signal was detected, by AFM measurements. Three monolayers, F14OH, F18OH and 20:80 F14OH + F18OH mixture were transferred onto silicon wafers by the Langmuir-Blodgett technique, at molecular areas within the coexistence plateau (0.7–0.85 nm^2^/molecule, *π* ~ 0 mN/m, *T* = 20 °C). Representative height images are presented in Figure 4.

These AFM images at large area per molecule clearly evidence the presence of solid-like aggregates, coexisting with a phase of very low density. As can be seen in the images, F14OH (cf. Figure 4a) aggregates as a network of round-edged entangled strings or threads, whereas F18OH forms mostly hexagon- or 6-pointed star-shaped polygonal structures (cf. Figure 4b). Under the same conditions, hydrogenated alcohols are well known to form circular liquid domains that coalesce upon compression. To the best of our knowledge, this is the first time that the spontaneous formation of such molecular aggregates is reported in Langmuir films of a pure perfluorinated alcohol.

The height profiles illustrate that, in both alcohols, the height of the visible domains is comparable to the length of the fully extended molecules (~1.8 nm for F14OH [44] and ~2.5 nm for F18OH [37]) within the experimental error. This is an indication that the molecules within the aggregates are perpendicular to the underlying substrate in agreement with the GIXD results. Remarkably, the film obtained from the F14OH + F18OH mixture shows aggregates of both morphologies (Figure 4c), displaying well-defined star-shaped structures, but now decorated with strings or threads. The height profiles of the two observed types of structures, measured on the mixed monolayer, are also similar, within uncertainty, to those obtained from the pure films. These observations suggest that, for the studied conditions (composition, *T*, *π* and *A*), the two alcohols condense separately in the mixed film, forming threads comprised mostly or exclusively of F14OH and star-shaped domains containing the remainder of the F18OH molecules.

### 3.4. Molecular Dynamics Simulations Results

Atomistic Molecular dynamics simulations were performed to further probe the behavior of the Langmuir films of the studied fluorinated alcohols. Simulations were performed for F12OH, F14OH, F16OH and F18OH to assess the influence of chain length on the behavior of the Langmuir films and at several areas per molecules, to obtain evidence of the various aggregation states of the fluorinated alcohols along the isotherm. For comparison, simulations were also carried out for hydrogenated alcohols of similar chain lengths.

**Figure 5:**
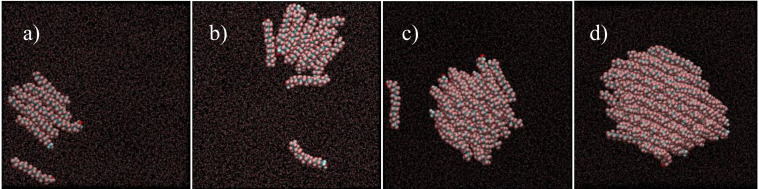
Representative top-view simulation snapshots of Langmuir films of F18OH at the surface of water, in the limit of very low surface density: (**a**) 8.28 nm^2^/molecule; (**b**) 6.37 nm^2^/molecule; (**c**) 2.59 nm^2^/molecule; (**d**) 1.29 nm^2^/molecule. The simulation boxes contain 10, 13, 32 and 64 alcohol molecules, respectively; water molecules are represented as dots for clarity.

**Figure 6:**
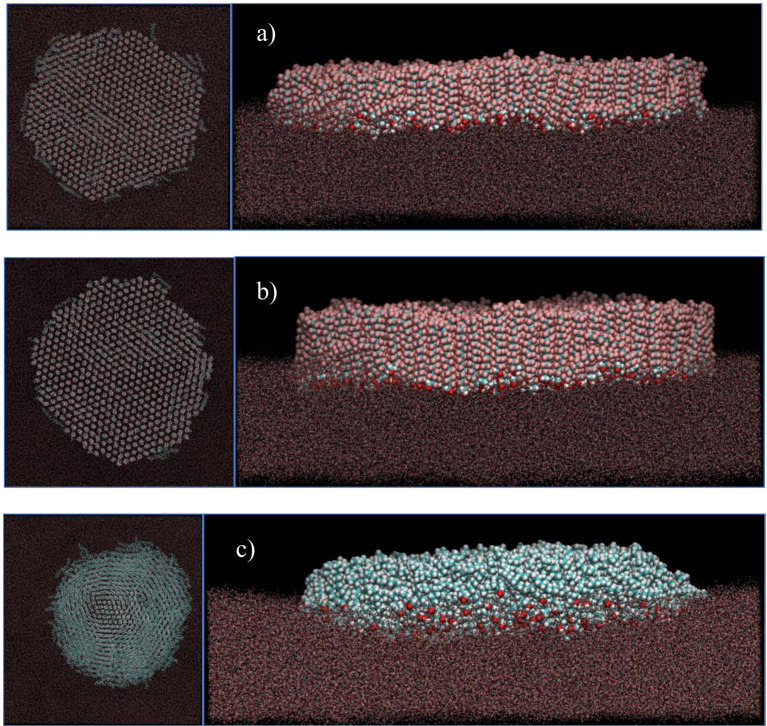
Representative snapshots, top-view (left) and side-view (right), of the equilibrated Langmuir films at the surface of water at 0.6 nm^2^/molecule of (**a**) F14OH, (**b**) F18OH and (**c**) H18OH. All films contain 550 alcohol molecules. Water molecules are represented by dots for clarity.

Figure 5 shows some representative snapshots of the equilibrated region of very low-surface density films (*A* between ~8.28 nm^2^/molecule and ~1.29 nm^2^/molecule) of F18OH. These simulations conducted along the *π* = 0 mN/m plateau show a gradual organization of the alcohol molecules along the compression isotherm. As can be seen, the fluorinated alcohol spontaneously forms aggregates at the surface of water, even for very small numbers of molecules at low surface density. In the smaller aggregates the alcohol molecules already tend to align parallel to each other, with most of the fluorinated chains in contact with the water surface. As the surface density increases (corresponding to an increase of the number of molecules in the simulation), the fluorinated chains start to form stacked structures, with the hydrophilic head in contact with water but increasing the lateral contact between the hydrophobic tails, which are nevertheless tilted towards the surface. The organization of the molecules within the aggregate increases with surface density, and in large enough aggregates most of the fluorinated chains are essentially perpendicular to the water surface, in a close hexagonal packing (Figure 6). It is important to notice that, at sufficiently low surface coverage, the aggregates are visibly in dynamic equilibrium with unbound and freely moving molecules, confirming that the used force field adequately models the coexistence of gaseous and condensed phases in agreement with the experimental evidence.

Larger aggregates comprising 550 molecules of F14OH and F18OH were also simulated at a surface density of 0.6 nm^2^/molecule. Representative snapshots are shown in Figure 6. An equivalent snapshot showing an aggregate of the hydrogenated alcohol H18OH is also included for comparison. It is apparent that both perfluorinated alcohols form much more organized domains that the hydrogenated alcohol H18OH, which displays an easily recognizable liquid-like structure. From the visualization of the snapshots and analysis of the simulation trajectory, it becomes evident that the F18OH molecules have a higher tendency for organization than F14OH, with better alignment between adjacent molecules and a smaller number of defects in the structure of the aggregate, which has better defined edges and a smaller number of molecules lying parallel to the surface of water.

**Figure 7:**
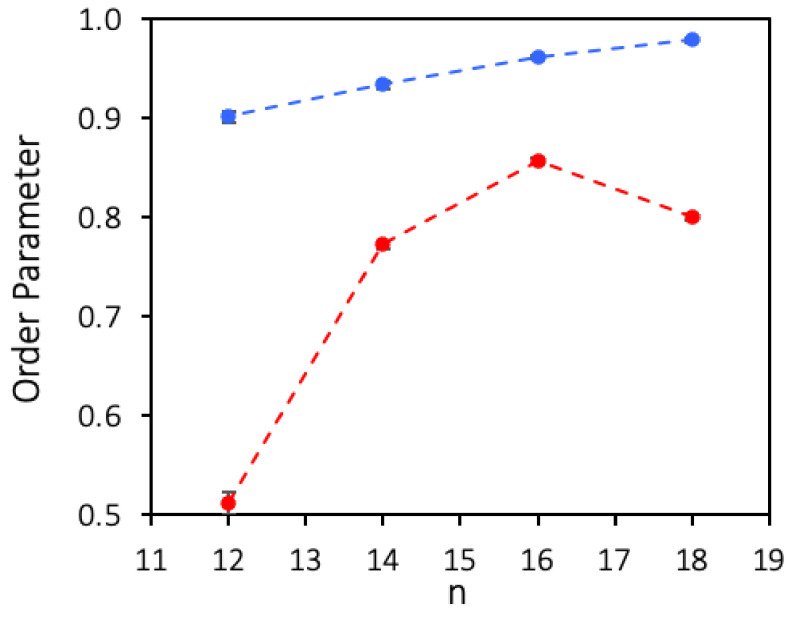
Simulated Average Order Parameter ($\hat{\mathrm{OP}}$) for Langmuir films at the surface of water of several fluorinated alcohols, F12OH, F14OH, F16OH and F18OH, and hydrogenated alcohols, H12OH, H14OH, H16OH and H18OH. All films contain 550 molecules at 0.6 nm^2^/molecule. The dashed lines are guides to the eye.

A quantitative comparison of the organization of the studied films was achieved calculating an Order Parameter (OP), based on the average of the dot product between all pairs of end-to-end vectors. The OP provides a first measure of how aligned the molecules are relative to one another, and is defined as:

(1) $$\mathrm{OP}=\frac{2}{N\left(N-1\right)}\overset{N-1}{\underset{i=1}{\sum }}\overset{N}{\underset{j>i}{\sum }}\frac{{\vec{r}}_{i}\left(t\right)\cdot {\vec{r}}_{j}\left(t\right)}{\left\|{\vec{r}}_{i}\left(t\right)\|\|{\vec{r}}_{j}\left(t\right)\right\|}$$

where ${\vec{r}}_{i}$ is the vector spanning molecule *i*, connecting the carbon atom bonded to the oxygen atom to the terminal carbon atom of the hydrophobic chain, $\|{\vec{r}}_{i}\left(t\right)∥$ is the modulus of vector ${\vec{r}}_{i}$, and the summations extend over the *N* alcohol molecules in the simulation box. If all the *N* molecules are aligned perfectly parallel to each other, OP assumes the value of 1, whereas lower values reflect lower degrees of organization. This parameter is calculated for each frame of the trajectory at time *t*, and its average over time ${\hat{\mathrm{OP}}}_{t}$ can be defined for a given simulation stretch. Figure 7 shows the values of $\hat{\mathrm{OP}}$ for films of several fluorinated and hydrogenated alcohols, with the corresponding standard deviations calculated by block averaging the equilibrated trajectories in 2 ns intervals.

As can be seen, all the studied perfluorinated alcohol films display values of $\hat{\mathrm{OP}}$ above 0.9, confirming the very high tendency of perfluorinated chains to pack laterally, with even the shorter molecules packing in essentially parallel fashion. From these results, it is also seen that $\hat{\mathrm{OP}}$ rises monotonically along the series, which can be explained either by a higher degree of parallel alignment of the longer molecules at the center of the aggregate, or by a lower number of longer molecules lying at the water surface near the aggregate’s rim. In the case of the hydrogenated alcohols, the order parameter is always considerably lower, increasing more slowly along the series. The OP of dodecanol is estimated to be much lower than the longer alcohols, in agreement with the experimentally determined low stability of the LB films of this alcohol.

**Figure 8:**
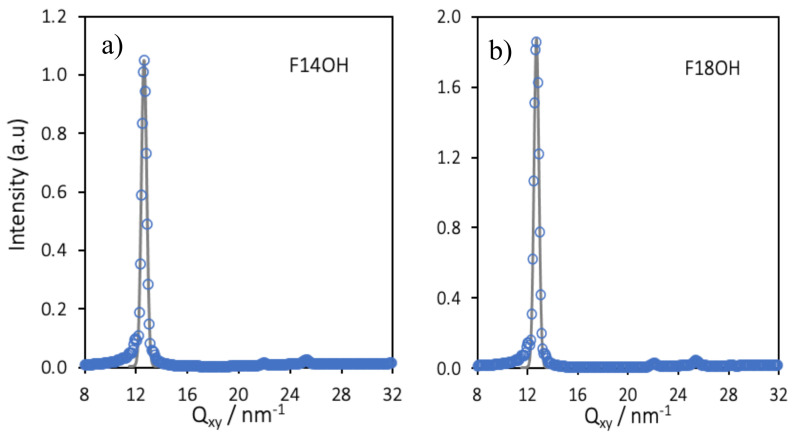
Simulated in-plane (Q_xy_) GIXD spectra of the 550 molecules Langmuir films of F14OH (**a**) and F18OH (**b**) at a molecular area of *A* = 0.6 nm^2^/molecule. Data points represent the calculated spectra and lines the Gaussian fits of the peaks.

Simulated in-plane (Q_xy_) GIXD patterns were calculated from the trajectories of the monolayers of F14OH and of F18OH at *A* = 0.6 nm^2^/molecule and the results are presented in Figure 8. Gaussian curves were fitted to the calculated GIXD patterns and are plotted alongside the calculated spectra. The positions of the diffraction peaks, obtained from the fitted curves, as well as the ratios of the positions of a few selected pairs of peaks are summarized in Table 4.

As can be seen in Table 4, the positions of the first diffraction peaks obtained from the simulations are very similar to those obtained experimentally, fully validating the simulation model and results. The underlying hexagonal molecular organization, already suggested by the visual representations in Figure 6, can be confirmed from the computed ratios Peak 2/Peak 1 and Peak/Peak 1, which are close to √3 and to 2, respectively. By indexing Peak 1 to the (10) peak of the hexagonal lattice, computed values of area per molecule of 0.286 nm^2^/molecule (F14OH) and 0.283 nm^2^/molecule (F18OH) are retrieved, in very good agreement with the values obtained from the experimental spectra. The simulation results, validated by the agreement between simulated and experimental GIXD spectra, provide further insights into the organization of the Langmuir films at the molecular scale.

## 4. Conclusions

Langmuir films of two highly fluorinated fatty alcohols, CF_3_(CF_2_)_12_CH_2_OH (F14OH) and CF_3_(CF_2_)_16_CH_2_OH (F18OH), and a (20:80) molar mixture of the two alcohols were studied. A set of experimental and computational techniques provides a complementary view of the organization and behavior of these systems at different length scales.

Atomic Force Microscopy (AFM) images of the films transferred at zero surface pressure onto the surface of silicon wafers by the Langmuir-Blodgett technique revealed, for the first time, the existence of solid-like domains with well-defined mostly hexagonal (starry) shapes in the case of F18OH, and with an entangled structure of strings and threads in the case of F14OH.

A (20:80) molar mixture of the two alcohols displayed a surprising combination of the two patterns characteristic of each alcohol: hexagonal domains decorated by zigzagging threads, clearly demonstrating that the two alcohols segregate during the 2D crystallization process.

Grazing Incidence X-ray Diffraction (GIXD) measurements confirmed that the molecules of both alcohols organize in 2D hexagonal lattices.

The star-shaped aggregates with hexagonal symmetry displayed by the F18OH Langmuir film are a manifestation in the micron range of that hexagonal molecular organization. It is likely that the film forms through a mechanism of nucleation and growth, as has been proposed for the formation of aggregates of partially fluorinated carboxylic acids at the air–water interface [51], although there are other similar systems whose formation is consistent with a 2D Ostwald ripening mechanism [69,70]. Notwithstanding, the mechanism of nucleation and growth would explain the existence of 6-pointed star-shaped domains: the “vertices” would then correspond to the preferential directions of aggregate growth.

Atomistic Molecular Dynamics (MD) simulations provide a visualization of the structure of the domains and allow a molecular-level interpretation of the experimental observations. The simulation results demonstrate that perfluorinated alcohols have an intrinsic tendency to aggregate at very low surface density, forming highly organized domains compared to those of hydrogenated alcohols with similar chain length. Thus, very probably, this tendency is a consequence of the characteristic stiffness of the perfluorinated chains. The diffraction spectrum calculated from the simulation trajectories compares favorably with the experimental spectra, fully validating the simulations and the proposed interpretation.

The present results highlight for the first time an inherent tendency of perfluorinated chains in monolayers to aggregate in highly organized 2D structures, even at very low surface density, in which fluorinated blocks upright as the surface density increases. We believe these findings are important to fully understand related phenomena, such as the formation of hemi-micelles of semifluorinated alkanes at the surface of water and the 2D segregation in mixed Langmuir films of hydrogenated and fluorinated fatty acids.

## Figures and Tables

**Table 1:** Limit molecular areas calculated from the *π*–*A* isotherms.

Molecule	Limit Molecular Area (Å^2^/molecule)
F18OH	27.6 ± 0.9
F14OH	31.2 ± 0.4
(20:80) F14OH + F18OH	27.0 ± 0.4

**Table 2:** Peak position (Q_xy_) and full width at half maximum (FWHM) of the diffraction peaks presented in Figure 2, obtained by fitting Lorentzian curves to the GIXD spectra.

Molecule	A (nm^2^/molecule)	Q_xy_ (nm^−1^)	FWHM (nm^−1^)	Hexagonal Lattice Parameter (nm)	Coherence Length (nm)
F14OH	0.60	12.62	0.020	0.575	>320
F18OH	0.45	12.65	0.029	0.5753	300

**Table 3:** Positions and full width at half maximum (FWHM) of the F18OH diffraction peaks at 15 mN/m obtained by fitting Gaussian curves.

Peak Indexation	Peak Position (nm^−1^)	FWHM (nm^−1^)
(10)	12.69	0.06
(11)	21.97	0.1
(20)	25.36	0.08

**Table 4:** Positions of the diffraction peaks and ratios of the positions of selected pairs of peaks, obtained for the simulated spectra presented in Figure 8.

Peak	Peak Position (nm^−1^)	
	F18OH	F14OH
Peak 1	12.69	12.62
Peak 2	22.06	21.98
Peak 3	25.40	25.25
Peak 2/Peak 1	1.738	1.741
Peak 3/Peak 1	2.001	2.001
